# Increasing beam stability zone in synchrotron light sources using polynomial quasi-invariants

**DOI:** 10.1038/s41598-023-27732-y

**Published:** 2023-01-24

**Authors:** Edgar Andrés Sánchez, Alain Flores, Jorge Hernández-Cobos, Matías Moreno, Armando Antillón

**Affiliations:** 1 Instituto de Ciencias Físicas, Universidad Nacional Autónoma de México, Av. Universidad 1001, Col. Chamilpa, Cuernavaca, Morelos 62210 Mexico; 2 Departamento de Bioingeniería y Ciencias, Tecnológico de Monterrey, Puebla, 72453 Mexico; 3 Departamento de Física Teórica, Instituto de Física, Universidad Nacional Autónoma de México, Cd. de México, 04510 Mexico

**Keywords:** Energy science and technology, Engineering, Mathematics and computing, Physics

## Abstract

The objective of this article is to propose a scheme to increase the stability zone of a charged particles beam in synchrotrons using a suitable objective function that, when optimized, inhibits the resonances onset in phase space and the dynamic aperture of electrons in storage rings can be improved. The proposed technique is implemented by constructing a quasi-invariant in a neighborhood of the origin of the phase space, then, by using symbolic computation software, sets of coupled differential equations for functions involved in nonlinear dynamics are obtained and solved numerically with periodic boundary conditions. The objective function is constructed by proposing that the innermost momentum solution branch of the polynomial quasi-invariant approaches to the corresponding ellipse of the linear dynamics. The objective function is optimized using a genetic algorithm, allowing the dynamic aperture to be increased. The quality of results obtained with this scheme are compared with particle tracking simulations performed with available software in the field, showing good agreement. The scheme is applied to a synchrotron light source model that can be classified as third generation due to its emittance.

## Introduction

Nowadays, the design of storage rings of synchrotron light sources is a major challenge, mainly because the dynamic aperture is reduced by non-linear properties of the lattice. In the first stage of the design, magnetic dipoles and quadrupoles are used to generate a linear achromatic lattice^1^ with a given emittance. The second step involves the introduction of magnetic sextupoles, and if needed, octupoles, which transform dynamics from linear to nonlinear, generating new phenomena that, if not controlled, are sources of instability of the electron beam. In such a case, the brilliance of the synchrotron radiation could be degraded affecting the ongoing experiments of technological, basic, and applied scientific research^2^.

When a synchrotron light source is operated, there can be several hundred electron bunches distributed along the ring. The bunches travel inside a metal tube at very high vacuum conditions, minimizing collisions with gas molecules. The tube passes through the center of all the magnets. The electron bunches must be stabilized by interacting with magnetic forces, provided by several magnetic multipoles. In operation, such stability must be guaranteed for several hours.

**Figure 1:**
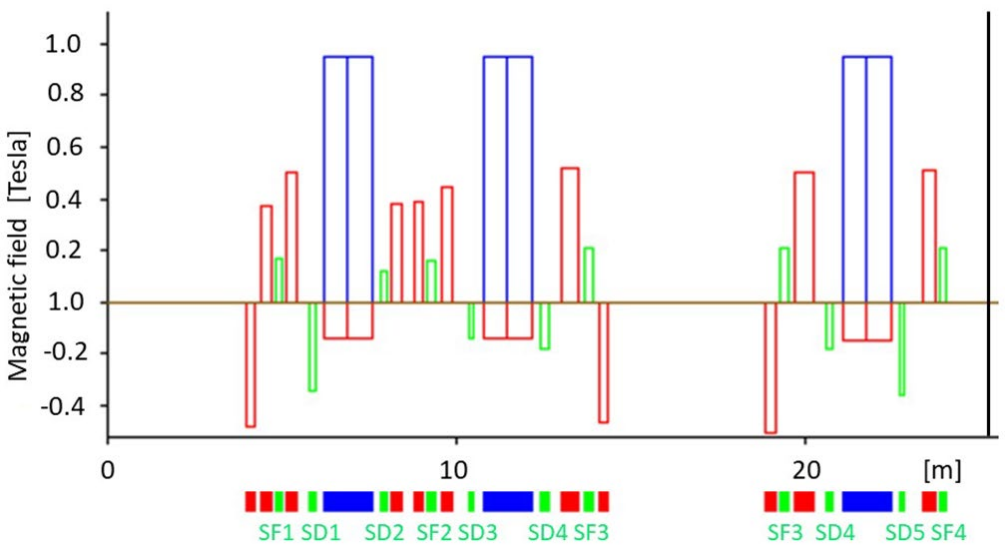
Magnetic fields of the first magnets in Fig. 2 are shown. In the lower part of the figure, the left set of magnets, separated by drift spaces, corresponds to a matching cell. The right set exhibits a half unit cell. The field strengths for the corresponding dipoles (blue), quadrupoles (red) and sextupoles (green) are depicted in the upper part of the figure. These field values are related with the parameters $b_n$ through Eq. (7).

**Figure 2:**
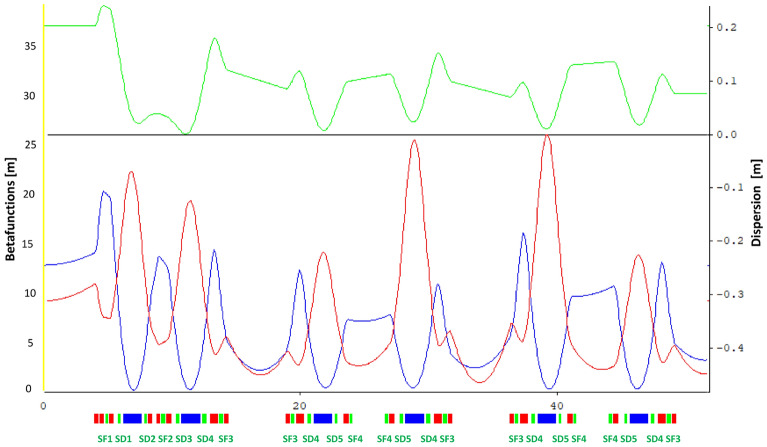
Optical functions of a half super period of the ALBA-like lattice. The functions $\beta _x(s)$ and $\beta _y(s)$ are marked in blue and red, respectively; and in green, the dispersion function $\eta (s)$. The lower part shows the distribution of dipoles (blue), quadrupoles (red) and sextupoles (green) of the three DBA-cells in a half super period. The lower green text line shows the assignment of the different sextupoles. The other half of the superperiod is symmetric about the vertical axis. The ring is composed of four superperiods.

When searching for a good design, it is necessary to optimize the different lengths of all magnets, their field strengths (the latter are described by the functions $b_1(s)$, $b_2(s)$, and $b_3(s)$ which are piecewise constant functions, as shown in Fig. 1), as well as the lengths of the free spaces between them, the so-called drift spaces. Usually, this process gives the ring a physical structure based on a periodic arrangement of magnetic cells, as indicated in Fig. 2. The process seeks for an arrangement of these magnets to allow electrons describe stable trajectories, traveling at speeds close to the speed of light.

In the continual search to reduce the emittance of new synchrotrons, the use of increasingly intense magnetic quadrupoles is required, giving rise to more noticeable chromatic effects. The use of high intensity magnetic sextupoles (chromatic), and higher order multipoles, is required to correct these effects. A different type of sextupoles, called geometric, is added to correct undesirable effects produced by chromatic sextupoles to improve the dynamic aperture. The more intense the sextupoles and higher order multipoles are, the more difficult it is to keep dynamic stability under control. At this level, the problem to be solved in the design process is to adjust the sextupoles families to simultaneously maximize both, the dynamic aperture, and the moment aperture. Additionally, the complexity increases if other types of important variables are included in the optimization^3^. Ultimately, the ring designs must be robust in the presence of non-linearities, intentional such as sextupoles, or unintentional, such as errors and imperfections in the field of the magnets. The conventional method to make these adjustments is to minimize many resonant terms^4,5^. These results are then validated with particle tracking simulation^6,7^. Moreover, it is common to use complementary tools such as frequency map analysis^8^ to have a better picture of the tune diffusion and resonance structures. Other effective optimization methods, very demanding in computational resources^9^, directly calculate the dynamic aperture by means of particle tracking^10–13^, including the calculation of resonant terms if required^14^. Nonlinear systems of great complexity in their analytical resolution procedures led some authors to analyze and develop novel methods to treat them numerically^12,13^. Some of those methods are based on highly accurate descriptive procedures^6,7^. Furthermore, the need to have machines with better performance has motivated researchers to propose new solutions such as integrable accelerators^15–17^, where the magnetic fields are modulated in such a way that integrals of motion are achieved.

It has also been suggested that averaged invariants could be useful to analyze the fully coupled dynamics over a single synchrotron oscillation^18^. There are also proposals for the use of approximate invariants, (or quasi-invariants)^19–22^, to advance the understanding of nonlinear dynamics in synchrotrons. Some of these proposals use particle tracking to make the system approximately integrable^20^.

Another proposed way of introducing approximate in- variants^23–25^, focusing on describing the phase space of design-friendly synchrotrons (such as a booster), seems appropriate to describe resonances appearing in the synchrotrons transverse phase space. These ideas had not been widely explored until recently, when an extension of this formalism to a 5th degree polynomial was applied to a 3rd. generation light source^26^. Our aim is to take a step forward from what has been presented in references^23–26^, supplying content and concepts that may be useful to understand nonlinear processes in synchrotrons and to improve synchrotron design performance. Now this formalism is employed to develop an algorithm for the exploration and manipulation of the phase space of these systems, focused on increasing the dynamic aperture of dynamically more complex synchrotrons. In this paper, an objective function, and an optimization scheme, based on it, are proposed to maximize the dynamic aperture of a synchrotron light-source. Although the optimization of the dynamic aperture of these systems using quasi-invariants has been addressed before^20^, the present work has a different approach and methodology. While Ref.^20^ uses particle tracking to make the system quasi-integrable, the present work proposes the search for a bounded stability zone using an objective function, forcing the phase space of the nonlinear system to resemble the linear one. This is based on the determination of the roots of a polynomial quasi-invariant, without using particle tracking nor considering the resonant terms. As far as we know, this approach has not been used before. In addition, this proposal has the advantage of locally considering, at a given approximation order, the possibility of inhibiting resonance onset. It will be shown that robust results are obtained as the amplitude of the oscillations increases and as the momentum dispersion is considered. In this way, it is shown that the proposed quasi-invariant based method can be a useful tool to increase the dynamic aperture of the electron beam in a synchrotron light source. Particle tracking methods (such as the one used by OPA) were used in the present work only for comparative purposes, showing a good agreement with the results obtained. A one-dimensional problem has been treated in this work, but it can be easily extended to 2D. This method provides a different strategy to address the problem of optimizing the dynamics of these systems and facilitate the design of new state-of-the-art synchrotron light sources.

## Approximate constants of motion

References^23–25^ describe a method to extend the linear theoretical structure to the nonlinear case in the study of synchrotron dynamics. There, the existence of nonlinear functions that play a similar role to that of functions $\alpha$, $\beta$, and $\gamma$ used in the linear case^27^ is proposed. With these nonlinear functions, quasi-invariants can be established, with validity in a local range of phase space.

Interest in quasi-invariants formalism continues^18^ because it could help in the design of modern particle accelerators,^28,29^, where the effects of nonlinear dynamics are increasingly important, adding complexity to the implementation of each design. Reducing these effects may contribute to have light sources with emittances that provide better quality and properties of the emitted light, enhancements that are very useful in cutting-edge experimental techniques in various areas of science.

### Approximate constants of motion for the one dimensional problem

Since there are 3 physical variables in synchrotron dynamics, the phase space of this system is 6-dimensional. Hence, in the long term, our objective is to address a more complex structure than the one presented here. However, constructing a more general formulation to address this complexity is not trivial, so it is very important to proceed in small but firm steps. In this way, a one-dimensional approximation is very useful for a deep understanding of dynamical problems of accelerators in lower dimensions. Similar approaches can be found in references^30–32^.

Following the structure of a previous work^26^, and for completeness of this work, one can consider a one-dimensional linear motion where the Hamiltonian has the form

1 $$\begin{aligned} H_0 = \frac{1}{2} \left( p_x^2 + K(s)\, x^2\right) , \end{aligned}$$

where ($x,p_x$) are canonical conjugate variables, $K (s) = K (s + c)$ is a periodic function of *s*, with period *c*, and represents the intensity of the magnetic quadrupoles of the storage ring. The red piece-wise constant function in Fig. 1 is related to function *K*(*s*). This system has the invariant

2 $$\begin{aligned} I_0 = \gamma _x(s)\, x^2 + 2 \alpha _x(s)\, x\, p_x + \beta _x(s)\, p_x^2, \end{aligned}$$

where $\alpha _x(s)$, $\beta _x(s)$ and $\gamma _x(s)$ are the periodic Courant-Snyder functions, also with period *c*^27^.

As reference^23^ shows, the above description can be extended to the one-dimensional nonlinear case described by the Hamiltonian

3 $$\begin{aligned} {\bar{H}} = \frac{1}{2}\left( p_x^2 + K(s)\, x^2\right) + S(s)\, x^3, \end{aligned}$$

In reference^23^, it is proposed that this system has an approximate invariant of the form

4 $$\begin{aligned} {\bar{I}} = \sum _{i+j\ge 2, i,j=0} A^{(0)}_{ij}(s) x^i p_x^j, \end{aligned}$$

where $A^{(0)}(s)=A^{(0)}(s+c)$ are periodic functions, which must satisfy differential equations imposed by the invariant condition

5 $$\begin{aligned} \frac{dI}{ds} = \{I,H\}+ \frac{\partial {I}}{\partial {s}}=0, \end{aligned}$$

and can be viewed as a generalization of the linear Courant-Snyder functions $\alpha _x$, $\beta _x$, and $\gamma _x$, for the nonlinear regime. Therefore, the proposed approximate invariant in Eq. (4) is a generalization of the linear Courant-Snyder invariant of Eq. (2). This idea has been proposed in references^23,25^ to treat chromatic effects in these systems, i.e., taking into account the possibility that the particles have a momentum *p* different from the design momentum $p_0$. It has been shown that when chromatic effects are included in this way, the results obtained are in good agreement with those obtained by simulations using numerical solutions of Hamilton equations.

The linear system (1) is relevant since its invariant (2) is well understood; thus, it is possible to compare the phase space structure of the nonlinear system with respect to the linear case by using an extension of the expression (2). Furthermore, this representation could be used to develop semi-analytical tools that can be useful in the nonlinear regime addressed in the accelerator design process.

This extension also allows the introduction of chromatic effects in the analytical framework, suggesting its usefulness as a complement within the analysis of Hamiltonian systems, as it will be discussed below.

### Searching for an approximate constant of motion for the two-dimensional problem

The approach to study electron dynamics in these systems usually consists of transforming the well-known relativistic Hamiltonian of a charged particle in an electromagnetic field, originally expressed in the laboratory system, to a moving reference system. After this change of coordinates, a transformation of variables is made allowing the longitudinal coordinate *s* to be the independent variable instead of the time *t*. From the resulting Hamiltonian, the equations of motion can be obtained; these calculations are standard, and their details can be seen in reference^4^. In certain circumstances the longitudinal motion (*s*, $p_s$) can be decoupled from the transverse motion (*x*, $p_x$, *y*, $p_y$), and it is even possible to decouple the transverse motion into a horizontal (*x*, $p_x$) and a vertical motions (*y*, $p_y$).

Let us use a more general expression of the Hamiltonian that appears in Eq. (3). Following the notation of references^4,33^, a Hamiltonian of the form

6 $$\begin{aligned} H(x,p_x,y,p_y,s)= & {} \frac{1}{2}(p_x^2 + p_y^2)\left( 1 - \delta + \delta ^2 + \cdots \right) \nonumber \\{} & {} - b_1(s) x \delta + \frac{b_1^2(s)}{2} x^2 + \frac{b_2(s)}{2} (x^2 - y^2) \nonumber \\{} & {} + \frac{b_3(s)}{3} (x^3 - 3xy^2) + \cdots , \end{aligned}$$

describes the transverse dynamics of particles in a synchrotron. This Hamiltonian has been widely used in preliminary dynamic aperture studies^4,34,35^, in the first stage of storage ring design.

The functions $$ b_1 (s)$$, $$ b_2 (s)$$, and $$ b_3 (s)$$ are periodic functions with piece-wise constant behavior, respectively describing the curvature of the dipoles and the intensities of the quadrupoles and sextupoles of the accelerator and $$\delta = \Delta p / p_0$$, where $$ \Delta p $$ is the momentum deviation with respect to design momentum $$p_0$$. $$b_1(s) = 1 / \rho $$ ($$\rho $$ is the radius of curvature of a particular dipole) and the remaining parameters are given by the following expression^34^

7 $$\begin{aligned} b_n(s) = \frac{1}{B\rho }\frac{1}{(n-1)!}\frac{\partial ^{n-1}B_y(x,y)}{\partial x^{n-1}}\mid _{y=0}, \end{aligned}$$

where $B\rho$ is the magnetic rigidity that connects magnetic field and radius of curvature with the energy of a relativistic electron via $B\rho [Tm] = 3.3356\, E[GeV]$. Also, if *R* is the pole inscribed radius, $b_n$ is related to the pole tip magnetic field $B_{pt}$ (Fig. 1) by the equation

8 $$\begin{aligned} B_{pt} = (B\rho )b_nR^{n-1}, \end{aligned}$$

that is very useful in determining fields for magnet design.

In references^24^ and^25^ it has been proposed that a quasi-invariant of the form

9 $$\begin{aligned} I=\sum _{i+j+k+l\ge 2, i,j,k,l=0} \sum _{n=0} A^{(n)}_{ijkl}(s) x^ip_x^jy^kp_y^l\delta ^n, \end{aligned}$$

can be associated with this Hamiltonian (Eq. (6)). Here, the chromatic nonlinear functions ($n \ge 1$) are also periodic, that is, $A^{(n)}_{ijkl}(s)=A^{(n)}_{ijkl}(s+c)$. When computed for a particular arc length *s*, these functions acquire numerical values.

Equation (9) represents a nonlinear extension of the Courant-Snyder invariant, Eq. (2); which means that to a second degree, the lowest order, only the *horizontal* functions $A^{(0)}_{ijkl}(s)$ are different from zero. The substitution of Eq. (9) in expression (5) leads to a system of linear differential equations. The number of equations depends on the polynomial degree considered. In this paper we consider a fifth-degree polynomial. The sixty meaningful associated differential equations corresponding to non-null, nonlinear functions related to on-momentum ($\delta = 0$) particles are presented below in Eqs. (10)–(18). The Eqs. (10)–(12) have already been considered in reference^26^.

10.1 $$\begin{aligned} \frac{d A^{(0)}_{2000}}{ds}&= \left( b_2 + b_1^{2}\right) A^{(0)}_{1100} \end{aligned}$$

10.2 $$\begin{aligned} \frac{d A^{(0)}_{1100}}{ds}&= - 2 A^{(0)}_{2000} + 2 \left( b_2 + b_1^{2}\right) A^{(0)}_{0200}\end{aligned}$$

10.3 $$\begin{aligned} \frac{d A^{(0)}_{0200}}{ds}&= - A^{(0)}_{1100} \end{aligned}$$

11.1 $$\begin{aligned} \frac{d A^{(0)}_{3000}}{ds}&= \left( b_2 + b_1^{2}\right) A^{(0)}_{2100} + b_3 A^{(0)}_{1100} \end{aligned}$$

11.2 $$\begin{aligned} \frac{d A^{(0)}_{2100}}{ds}&= -3 A^{(0)}_{3000} + 2 \left( b_2 + b_1^{2}\right) A^{(0)}_{1200} + 2 b_3 A^{(0)}_{0200} \end{aligned}$$

11.3 $$\begin{aligned} \frac{d A^{(0)}_{1200}}{ds}&= -2 A^{(0)}_{2100} + 3 \left( b_2 + b_1^{2}\right) A^{(0)}_{0300} \end{aligned}$$

11.4 $$\begin{aligned} \frac{d A^{(0)}_{0300}}{ds}&= - A^{(0)}_{1200} \end{aligned}$$

12.1 $$\begin{aligned} \frac{d A^{(0)}_{1020}}{ds}&= - b_3 A^{(0)}_{1100} - b_2 A^{(0)}_{1011} + \left( b_2 + b_1^{2}\right) A^{(0)}_{0120} \end{aligned}$$

12.2 $$\begin{aligned} \frac{d A^{(0)}_{1011}}{ds}&= - 2 A^{(0)}_{1020} - 2 b_2 A^{(0)}_{1002} + \left( b_2 + b_1^{2}\right) A^{(0)}_{0111} \end{aligned}$$

12.3 $$\begin{aligned} \frac{d A^{(0)}_{0120}}{ds}&= - A^{(0)}_{1020} - 2 b_3 A^{(0)}_{0200} - b_2 A^{(0)}_{0111} \end{aligned}$$

12.4 $$\begin{aligned} \frac{d A^{(0)}_{0111}}{ds}&= - A^{(0)}_{1011} - 2 A^{(0)}_{0120} - 2 b_2 A^{(0)}_{0102} \end{aligned}$$

12.5 $$\begin{aligned} \frac{d A^{(0)}_{1002}}{ds}&= - A^{(0)}_{1011} + \left( b_2 + b_1^{2}\right) A^{(0)}_{0102}\end{aligned}$$

12.6 $$\begin{aligned} \frac{d A^{(0)}_{0102}}{ds}&= - A^{(0)}_{1002} - A^{(0)}_{0111} \end{aligned}$$

13.1 $$\begin{aligned} \frac{d A^{(0)}_{4000}}{ds}&= b_2 A^{(0)}_{3100}+b_1^2 A^{(0)}_{3100}+b_3 A^{(0)}_{2100}\end{aligned}$$

13.2 $$\begin{aligned} \frac{d A^{(0)}_{3100}}{ds}&= -4 A^{(0)}_{4000}+2 b_2 A^{(0)}_{2200}+2 b_1^2 A^{(0)}_{2200}\nonumber \\ {}&+2 b_3 A^{(0)}_{1200}\end{aligned}$$

13.3 $$\begin{aligned} \frac{d A^{(0)}_{2200}}{ds}&= -3 A^{(0)}_{3100}+3 b_2 A^{(0)}_{1300}+3 b_1^2 A^{(0)}_{1300}\nonumber \\ {}&+3 b_3 A^{(0)}_{0300}\end{aligned}$$

13.4 $$\begin{aligned} \frac{d A^{(0)}_{1300}}{ds}&= -2 A^{(0)}_{2200}+4 b_2 A^{(0)}_{0400}+4 b_1^2 A^{(0)}_{0400}\end{aligned}$$

13.5 $$\begin{aligned} \frac{d A^{(0)}_{0400}}{ds}&= -A^{(0)}_{1300} \end{aligned}$$

14.1 $$\begin{aligned} \frac{d A^{(0)}_{2020}}{ds}&= -b_3 A^{(0)}_{2100}-b_2 A^{(0)}_{2011}+b_2 A^{(0)}_{1120}+b_1^2 A^{(0)}_{1120}\nonumber \\&-2 b_3 A^{(0)}_{1011}+b_3 A^{(0)}_{0120}\end{aligned}$$

14.2 $$\begin{aligned} \frac{d A^{(0)}_{2011}}{ds}&= -2 A^{(0)}_{2020}-2 b_2 A^{(0)}_{2002}+b_2 A^{(0)}_{1111}+b_1^2 A^{(0)}_{1111}\nonumber \\&-4 b_3 A^{(0)}_{1002}+b_3 A^{(0)}_{0111}\end{aligned}$$

14.3 $$\begin{aligned} \frac{d A^{(0)}_{1120}}{ds}&= -2 A^{(0)}_{2020}-2 b_3 A^{(0)}_{1200}-b_2 A^{(0)}_{1111}+2 b_2 A^{(0)}_{0220}\nonumber \\&+2 b_1^2 A^{(0)}_{0220}-2 b_3 A^{(0)}_{0111}\end{aligned}$$

14.4 $$\begin{aligned} \frac{d A^{(0)}_{2002}}{ds}&= -A^{(0)}_{2011}+b_2 A^{(0)}_{1102}+b_1^2 A^{(0)}_{1102}+b_3 A^{(0)}_{0102}\end{aligned}$$

14.5 $$\begin{aligned} \frac{d A^{(0)}_{1111}}{ds}&= -2 A^{(0)}_{2011}-2 A^{(0)}_{1120}-2 b_2 A^{(0)}_{1102}+2 b_2 A^{(0)}_{0211}\nonumber \\&+2 b_1^2 A^{(0)}_{0211}-4 b_3 A^{(0)}_{0102}\end{aligned}$$

14.6 $$\begin{aligned} \frac{d A^{(0)}_{0211}}{ds}&= -A^{(0)}_{1111}-2 A^{(0)}_{0220}-2 b_2 A^{(0)}_{0202}\end{aligned}$$

14.7 $$\begin{aligned} \frac{d A^{(0)}_{0220}}{ds}&= -A^{(0)}_{1120}-3 b_3 A^{(0)}_{0300}-b_2 A^{(0)}_{0211} \end{aligned}$$

14.8 $$\begin{aligned} \frac{d A^{(0)}_{1102}}{ds}&= -2A^{(0)}_{2002}-A^{(0)}_{1111}+2 b_2 A^{(0)}_{0202}+2 b_1^2 A^{(0)}_{0202}\end{aligned}$$

14.9 $$\begin{aligned} \frac{d A^{(0)}_{0202}}{ds}&= -A^{(0)}_{1102}-A^{(0)}_{0211} \end{aligned}$$

15.1 $$\begin{aligned} \frac{d A^{(0)}_{0040}}{ds}&= -b_3 A^{(0)}_{0120}-b_2 A^{(0)}_{0031}\end{aligned}$$

15.2 $$\begin{aligned} \frac{d A^{(0)}_{0031}}{ds}&= -b_3 A^{(0)}_{0111}-4 A^{(0)}_{0040}-2 b_2 A^{(0)}_{0022} \end{aligned}$$

15.3 $$\begin{aligned} \frac{d A^{(0)}_{0022}}{ds}&= -b_3 A^{(0)}_{0102}-3 A^{(0)}_{0031}-3 b_2 A^{(0)}_{0013}\end{aligned}$$

15.4 $$\begin{aligned} \frac{d A^{(0)}_{0013}}{ds}&= -2 A^{(0)}_{0022}-4 b_2 A^{(0)}_{0004}\end{aligned}$$

15.5 $$\begin{aligned} \frac{d A^{(0)}_{0004}}{ds}&= -A^{(0)}_{0013} \end{aligned}$$

16.1 $$\begin{aligned} \frac{d A^{(0)}_{5000}}{ds}&= b_2 A^{(0)}_{4100}+b_1^2 A^{(0)}_{4100}+b_3 A^{(0)}_{3100}\end{aligned}$$

16.2 $$\begin{aligned} \frac{d A^{(0)}_{4100}}{ds}&= -5 A^{(0)}_{5000}+2 b_2 A^{(0)}_{3200}+2 b_1^2 A^{(0)}_{3200}+2 b_3 A^{(0)}_{2200}\end{aligned}$$

16.3 $$\begin{aligned} \frac{d A^{(0)}_{3200}}{ds}&= -4 A^{(0)}_{4100}+3 b_2 A^{(0)}_{2300}+3 
b_1^2 A^{(0)}_{2300}+3 b_3 A^{(0)}_{1300} \end{aligned}$$

16.4 $$\begin{aligned} \frac{d A^{(0)}_{2300}}{ds}&= -3 A^{(0)}_{3200}+4 b_2 A^{(0)}_{1400}+4 b_1^2 A^{(0)}_{1400}+4 b_3 A^{(0)}_{0400} \end{aligned}$$

16.5 $$\begin{aligned} \frac{d A^{(0)}_{1400}}{ds}&= -2 A^{(0)}_{2300}+5 b_2 A^{(0)}_{0500}+5 b_1^2 A^{(0)}_{0500}\end{aligned}$$

16.6 $$\begin{aligned} \frac{d A^{(0)}_{0500}}{ds}&= -A^{(0)}_{1400} \end{aligned}$$

17.1 $$\begin{aligned} \frac{d A^{(0)}_{3020}}{ds}&= -b_3 A^{(0)}_{3100}-b_2 A^{(0)}_{3011}+b_2 A^{(0)}_{2120}+b_1^2 A^{(0)}_{2120}\nonumber \\&-2 b_3 A^{(0)}_{2011}+b_3 A^{(0)}_{1120}\end{aligned}$$

17.2 $$\begin{aligned} \frac{d A^{(0)}_{3011}}{ds}&= -2 A^{(0)}_{3020}-2 b_2 A^{(0)}_{3002}+b_2 A^{(0)}_{2111}+b_1^2 A^{(0)}_{2111}\nonumber \\&-4 b_3 A^{(0)}_{2002}+b_3 A^{(0)}_{1111}\end{aligned}$$

17.3 $$\begin{aligned} \frac{d A^{(0)}_{2120}}{ds}&= -3 A^{(0)}_{3020}-2 b_3 A^{(0)}_{2200}-b_2 A^{(0)}_{2111}+2 b_2 A^{(0)}_{1220}\nonumber \\&+2 b_1^2 A^{(0)}_{1220}-2 b_3 A^{(0)}_{1111}+2 b_3 A^{(0)}_{0220}\end{aligned}$$

17.4 $$\begin{aligned} \frac{d A^{(0)}_{3002}}{ds}&= -A^{(0)}_{3011}+b_2 A^{(0)}_{2102}+b_1^2 A^{(0)}_{2102}+b_3 A^{(0)}_{1102}\end{aligned}$$

17.5 $$\begin{aligned} \frac{d A^{(0)}_{2111}}{ds}&= -3 A^{(0)}_{3011}-2 A^{(0)}_{2120}-2 b_2 A^{(0)}_{2102}+2 b_2 A^{(0)}_{1211}\nonumber \\&+2 b_1^2 A^{(0)}_{1211}-4 b_3 A^{(0)}_{1102}+2 b_3 A^{(0)}_{0211}\end{aligned}$$

17.6 $$\begin{aligned} \frac{d A^{(0)}_{1220}}{ds}&= -2 A^{(0)}_{2120}-3 b_3 A^{(0)}_{1300}-b_2 A^{(0)}_{1211}+3 b_2 A^{(0)}_{0320}\nonumber \\&+3 b_1^2 A^{(0)}_{0320}-2 b_3 A^{(0)}_{0211}\end{aligned}$$

17.7 $$\begin{aligned} \frac{d A^{(0)}_{2102}}{ds}&= -3 A^{(0)}_{3002}-A^{(0)}_{2111}+2 b_2 A^{(0)}_{1202}+2 b_1^2 A^{(0)}_{1202}\nonumber \\&+2 b_3 A^{(0)}_{0202} \end{aligned}$$

17.8 $$\begin{aligned} \frac{d A^{(0)}_{1211}}{ds}&= -2 A^{(0)}_{2111}-2 A^{(0)}_{1220}-2 b_2 A^{(0)}_{1202}+3 b_2 A^{(0)}_{0311}\nonumber \\&+3 b_1^2 A^{(0)}_{0311}-4 b_3 A^{(0)}_{0202} \end{aligned}$$

17.9 $$\begin{aligned} \frac{d A^{(0)}_{0320}}{ds}&= -A^{(0)}_{1220}-4 b_3 A^{(0)}_{0400}-b_2 A^{(0)}_{0311} \end{aligned}$$

17.10 $$\begin{aligned} \frac{d A^{(0)}_{1202}}{ds}&= -2 A^{(0)}_{2102}-A^{(0)}_{1211}+3 b_2 A^{(0)}_{0302}+3 b_1^2 A^{(0)}_{0302} \end{aligned}$$

17.11 $$\begin{aligned} \frac{d A^{(0)}_{0311}}{ds}&= -A^{(0)}_{1211}-2 A^{(0)}_{0320}-2 b_2 A^{(0)}_{0302} \end{aligned}$$

17.12 $$\begin{aligned} \frac{d A^{(0)}_{0302}}{ds}&= -A^{(0)}_{1202}-A^{(0)}_{0311} \end{aligned}$$

18.1 $$\begin{aligned} \frac{d A^{(0)}_{1040}}{ds}&= -b_3 A^{(0)}_{1120}-b_2 A^{(0)}_{1031}+b_2 A^{(0)}_{0140}+b_1^2 A^{(0)}_{0140}\nonumber \\&-2 b_3 A^{(0)}_{0031}\end{aligned}$$

18.2 $$\begin{aligned} \frac{d A^{(0)}_{1031}}{ds}&= -b_3 A^{(0)}_{1111}-4 A^{(0)}_{1040}-2 b_2 A^{(0)}_{1022}+b_2 A^{(0)}_{0131}\nonumber \\&+b_1^2 A^{(0)}_{0131}-4 b_3 A^{(0)}_{0022}\end{aligned}$$

18.3 $$\begin{aligned} \frac{d A^{(0)}_{0140}}{ds}&= -A^{(0)}_{1040}-2 b_3 A^{(0)}_{0220}-b_2 A^{(0)}_{0131}\end{aligned}$$

18.4 $$\begin{aligned} \frac{d A^{(0)}_{1022}}{ds}&= -b_3 A^{(0)}_{1102}-3 A^{(0)}_{1031}-3 b_2 A^{(0)}_{1013}+b_2 A^{(0)}_{0122}\nonumber \\&+b_1^2 A^{(0)}_{0122}-6 b_3 A^{(0)}_{0013}\end{aligned}$$

18.5 $$\begin{aligned} \frac{d A^{(0)}_{0131}}{ds}&= -A^{(0)}_{1031}-2 b_3 A^{(0)}_{0211}-4 A^{(0)}_{0140}-2 b_2 A^{(0)}_{0122} \end{aligned}$$

18.6 $$\begin{aligned} \frac{d A^{(0)}_{1013}}{ds}&= -2 A^{(0)}_{1022}-4 b_2 A^{(0)}_{1004}+b_2 A^{(0)}_{0113}+b_1^2 A^{(0)}_{0113}\nonumber \\&-8 b_3 A^{(0)}_{0004} \end{aligned}$$

18.7 $$\begin{aligned} \frac{d A^{(0)}_{0122}}{ds}&= -A^{(0)}_{1022}-2 b_3 A^{(0)}_{0202}-3 A^{(0)}_{0131}-3 b_2 A^{(0)}_{0113}\end{aligned}$$

18.8 $$\begin{aligned} \frac{d A^{(0)}_{1004}}{ds}&= -A^{(0)}_{1013}+b_2 A^{(0)}_{0104}+b_1^2 A^{(0)}_{0104} \end{aligned}$$

18.9 $$\begin{aligned} \frac{d A^{(0)}_{0113}}{ds}&= -A^{(0)}_{1013}-2 A^{(0)}_{0122}-4 b_2 A^{(0)}_{0104}\end{aligned}$$

18.10 $$\begin{aligned} \frac{d A^{(0)}_{0104}}{ds}&= -A^{(0)}_{1004}-A^{(0)}_{0113}. \end{aligned}$$

Although equations for the non-linear functions $A^{(1)}_{ijkl}(s)$ for $\delta \ne 0$ were presented in Ref.^26^, that can be implemented for treating off-momentum particles, we have only used $A^{(0)}_{ijkl}(s)$ (on-momentum) in order to investigate the suitability of the proposed approach to produce good results when optimizing dynamic aperture. An extension to off-momentum analysis is underway.

It is possible to incorporate the longitudinal motion into a more general Hamiltonian and reapply the quasi-invariant protocol in 6D space, as is done using other methods (TPSA/tracking)^36^. This, however, is beyond the scope of this work as the 4D system has not yet been developed.

The differential equations (10-18) for the $A's$ functions, and the periodicity conditions $A(s+c) = A(s)$, imposed by the considered magnetic lattice (the specific selection of magnetic multipoles in the ring), determine the values of the functions *A*(*s*) in a period, which is usually a synchrotron cell. The synchrotron ring consists of an assembly of several of these cells.

Note that expressions (10) reproduce the equations satisfied by the Courant-Snyder parameters $\alpha _x$, $\beta _x$ and $\gamma _x$, inherent to linear dynamics; while Eqs. (11) and (12) are the equations of the first non-null nonlinear functions in the expansion (Eq. (9)), which must be satisfied for invariant condition (Eq. (5)) to be fulfilled. Equation system (11) only involves functions associated with the horizontal motion *x* in the accelerator, while the functions of equation system (12) couple the horizontal and vertical motions. In all the equation systems there are only functions $A^{(0)}$, which describe on-momentum particles. The algebraic manipulation to obtain these equations has been carried out with wxMaxima^37^.

Another point that deserves attention is that the set of functions in Eq. (10): $A^{(0)}_{2000}$, $A^{(0)}_{1100}$ and $A^{(0)}_{0200}$ are of essential importance. Real and bounded values of these functions are possible thanks to an appropriate selection of quadrupoles in the lattice, whereas the use of inappropriate values would lead to instability of the linear solution. The existence of non-zero values of higher order functions, like those appearing in Eqs. (11) and (12), occurs thanks to the fact that the aforementioned functions intervene in the non-homogeneous part of the equations, giving them nonzero values. Analogously, in those equations that contain nonlinear functions, the sextupolar contribution $b_3(s)$ appears for the first time. If this contribution were zero, the functions contained in Eqs. (11) and (12) must be zero, i.e., the quasi-invariant Eq. (9) will be reduced to the Courant-Snyder invariant of Eq. (2).

Reference^26^ outlines the procedure to determine the sixty values of the functions $A^{(0)}(s)$, at $s=0$, appearing in Eqs. (10)–(18), subjected to periodic boundary conditions $A^{(0)}_{ijkl}(0)=A^{(0)}_{ijkl}(0+c)$.

Only the quasi-invariant associated with horizontal motion has been studied in this work since the oscillation amplitudes in the horizontal plane are larger than those in the vertical plane; therefore, dynamics in the horizontal phase space is more complex than in the vertical phase space.

### An analytical approximate representation for Poincaré sections through quasi-invariants

It is well known that the quadrupolar magnetic components (represented in general by $$ b_2(s))$$ contained in an accelerator affect the off-momentum particles ($$ p_0 + \Delta p $$) differently from the reference particle with momentum $$p_0$$. This changes the number of oscillations in the horizontal and vertical planes (tunes $$\nu _x$$, $$\nu _y$$), deviating them from the original tunes $$\nu _{0x}$$, $$\nu _{0y}$$, in the following way

19 $$\begin{aligned} \nu _{x,y}(\delta )= \nu _{0x,y} + \xi _{x,y}\ \delta + \cdots \end{aligned}$$

where $\xi _{x,y}$, the horizontal and vertical chromaticities, are given by expression (20) taking $b_3(s)=0$.

In a bunch, particles have energies that differ from the energy of design. Therefore, quadrupoles in the accelerator supply a focusing force to each particle that depends on its energy, originating a chromatic effect. Furthermore, any kind of field imperfections in the quadrupoles will cause the same effect on electrons traveling in their orbits (see Ref.^38^).

Stability of the off-momentum particles requires that $\xi _x$ and $\xi _y$ be close to zero to keep tunes near to the operating point, while small positive values are required to avoid collective resonances. To satisfy these requirements, it is necessary to introduce magnetic sextupoles $b_3(s)$ in the lattice. Sextupoles affect chromaticities in the form^34,39–42^

20 $$\begin{aligned} \xi _{x,y} = \mp \frac{1}{4\pi } \int _{s_0}^{s_0+C} {\left[ b_2(s) - 2 b_3(s) \eta (s)\right] \beta _{x,y}(s) ds}. \end{aligned}$$

The function $\beta _y(s)$ plays a similar role for the vertical motion as $\beta _x(s)$ does for the horizontal case, and *C* is the circumference of full storage ring. $\eta (s)$ is known as dispersion function and it quantifies how much off-momentum particles closed orbit differs from on-momentum particles closed orbit.

## Application of the quasi-invariant technique to a third-generation synchrotron

Synchrotron light sources are classified into generations, based on the properties of the emitted light and the devices used to produce it. The complexity of beam dynamics increases with each generation, in such a way that new facilities demand better optimization techniques for a superior performance. In this work, a third-generation typical lattice is used to study some relevant aspects of our quasi-invariant method (see also^23–25^), since our main objective is to present its advantages.

### Application of quasi-invariants to a toy model for the Mexican synchrotron

The storage ring model presented below was, at some point, considered a low-risk scheme for the Mexican project^43^. It is based on the ALBA lattice^44^ and due to its emittance value, 1.3 nm$\cdot$rad, can be considered as a third-generation light source. Its linear optic functions are shown in Fig. 2 for one half super period. DBA-cells are shown at the bottom of the figure, where dipoles, quadrupoles, and sextupoles are depicted in blue, red, and green, respectively. The complete ring has 4 super periods; each super period consists of six DBA-cells: two matching cells at the ends and four-unit cells in the middle. In Fig. 1 the whole matching cell and half a unit cell are shown in more detail, depicting the magnetic fields of each magnet, following the magnets color code.

Once the dipole and quadrupole magnetic fields have been fixed, the storage ring optical functions and main parameters are determined; the latter are shown in Table 1. Natural chromaticities indicate that the sextupole intensities should not be very large, therefore, when performing chromaticity corrections, undesirable nonlinear phenomena should be minimal. We used this simple lattice to show the capability of the quasi-invariant formalism to inhibit the onset of resonances and to increase the dynamic aperture of synchrotrons.

**Table 1:** Main parameters of the storage ring shown in Fig. 2.

Parameter	Value
Energy	$3 \; \textrm{GeV}$
Circumference	$411.9 \; \textrm{m}$
super periods	$4 \;$
Betatron tunes $(\nu _x, \nu _y)$	27.12, 14.88
Natural chromaticities $(\xi _x, \xi _y)$	$-62.28, -38.74$
Emittance $(\epsilon _x)$	$1.33 \; \textrm{nm} \cdot \textrm{rad}$
Moment compaction factor $(\alpha _c)$	$3.9 \times 10 ^ {- 4}$

### Manipulating the onset of low order resonances to increase the dynamic aperture

The mechanism to find the roots of the polynomial quasi-invariant was described in refs.^23,26^, however, for the sake of completeness of this work, the methodology to find the points ($x,p_x$) that belong to a value of the quasi-invariant of Eq. (9), is outlined below. For $p_x=0$, the magnitude of the initial oscillation amplitude $x_0$, a numerical value of the quasi-invariant is obtained through the Courant-Snyder expression as $I=x_0^2/\gamma _x$; in our case ($\alpha _x=0$ since $s_0=0$ is a point of symmetry in the lattice). The numerical value of *I* is kept for the non-linear case and then the topological deformation of the phase space is calculated. Then, scanning *x*, for each value of *x*, the 5 values of $p_x$ (roots of the polynomial of degree 5) are found, which can be complex by pairs or real, using the root-finding function *roots* from $\hbox {MATLAB}^{\circledR}$. In this way, a set of points $(x,p_{x1}),...,(x,p_{x5})$ is obtained for a specific value of the quasi-invariant *I*. By plotting these pairs ($x, p_x$) in phase space, a sequence of nearby points is obtained that visually appear to form (interpolating) a continuous curve. These sequences will form what we have called a branch.

When a pair of branches overlap, the overlapping zone contains complex roots, and the non-overlapping portions will form islands indicating the presence of resonances. If there is no such overlap the resonances will not appear, as described in Ref.^26^. A similar procedure could be carried out by scanning $p_x$ and finding the values of the *x* roots of the quasi-invariant polynomial.

Once the process of resonances onset has been identified, we are interested in avoiding resonance formation while increasing the oscillation amplitude. With this, the method can provide a mechanism to increase the dynamic aperture of the synchrotron under consideration. A protocol is required to handle the roots in a controlled manner as the nonlinear elements are changed by the numerical optimization of the objective function, Eq. (21).

One way to do this is to separate the real root branches that give rise to resonances while increasing the amplitude of the horizontal oscillation. This is largely achieved by requiring that the innermost branches, which in principle form the deformed ellipse, have the best resemblance to the ellipse that arises when nonlinear elements (sextupoles, octupoles, etc.) are not present in the calculus, i.e., linear dynamics. With this procedure, a nonlinear phase space is forced to resemble a linear one by finding near-integrable solutions in a bound area of small amplitudes.

### The proposal of an objective function

A simple way to define an objective function is to quantify the separation between the branches of real roots corresponding to the nonlinear problem {$p_x^{nl}\}$ and the branches of real roots corresponding to the linear problem {$p_x^{ l}\}$; that is, the upper and lower branches of an ellipse that are obtained for the reference particle, when only dipoles and quadrupoles are considered. If we consider *N* points on the *x* axis, the corresponding lower and upper roots are in abbreviated form, {$p_x(x_i), i=1\cdot \cdot \cdot N\}$. Then, the 2*N* distances between the upper branches (*u*) and the lower branches (*d*) of the two systems, linear and nonlinear, are added to define the objective function $f_{obj}$ as

21 $$\begin{aligned} f_{obj}= \sum _{i=1}^{i=N}{\mid {p_x^{nl}(x_i)-p_x^l(x_i)\mid _{u}}}+\sum _{i=1}^{i=N}{\mid {p_x^{nl}(x_i)-p_x^l(x_i)\mid _{d}}}. \end{aligned}$$

Minimizing $f_{obj}$, we will be looking for solutions of the nonlinear problem that are as close as possible to the linear problem. As mentioned before, the nonlinear phase space is intended to resemble that of the linear case for small amplitudes.

The definition of $f_{obj}$ in Eq. (21) is done for the on-momentum reference particle, i.e, at $\delta =0$, but it can be extended to include terms corresponding to off-momentum particles ($\delta \ne 0$).

The objective function defined in the expression (21) considers the sum of point-to-point distances between the linear and non-linear trajectories in phase space. Since these distances depend on the nonlinear functions $A^{0}(s)$, the terms that most deform the phase space will be penalized the most. Being the only function that is intended to be optimized, the inclusion of weights in the problem is not necessary. We consider this to be a strength of the algorithm and the use of the objective function of Eq. (21).

### Initial stage

Defining $S=b_3/3$, the free parameters to optimize are

22 $$\begin{aligned} \{S\}_{free}=\{SD2, SD3, SD4, SD5, SF2, SF3, SF4\}, \end{aligned}$$

while the sextupoles

23 $$\begin{aligned} \{S\}_{chrom}=\{SD1, SF1\} \end{aligned}$$

are adjusted at each change made to $\{S\}_{free}$, by the optimization algorithm, in order to keep the chromaticities close to zero, $\xi _{x,y} \sim 0$.

Let us write the union of the above sets simply as

24 $$\begin{aligned} \{S\} = \{S\}_{free} \cup \{S\}_{chrom}. \end{aligned}$$

The initial set $S_0$ comes from the first attempts to confirm that the quasi-invariant method gave reliable results and compatible with particle tracking simulation. It was used in Ref.^26^ and has the following numerical values

25 $$\begin{aligned} \{S_0\}=&\{-5.3788110533658759, 1.3839068709904867,\nonumber \\ {}&-8.8951634707862990, -15.453045920930355,\nonumber \\ {}&21.482198067784918, 1.2586848537899471,\nonumber \\&11.837997264320983, -17.378852082005590,\nonumber \\ {}&19.49703775940865 \} \end{aligned}$$

**Figure 3:**
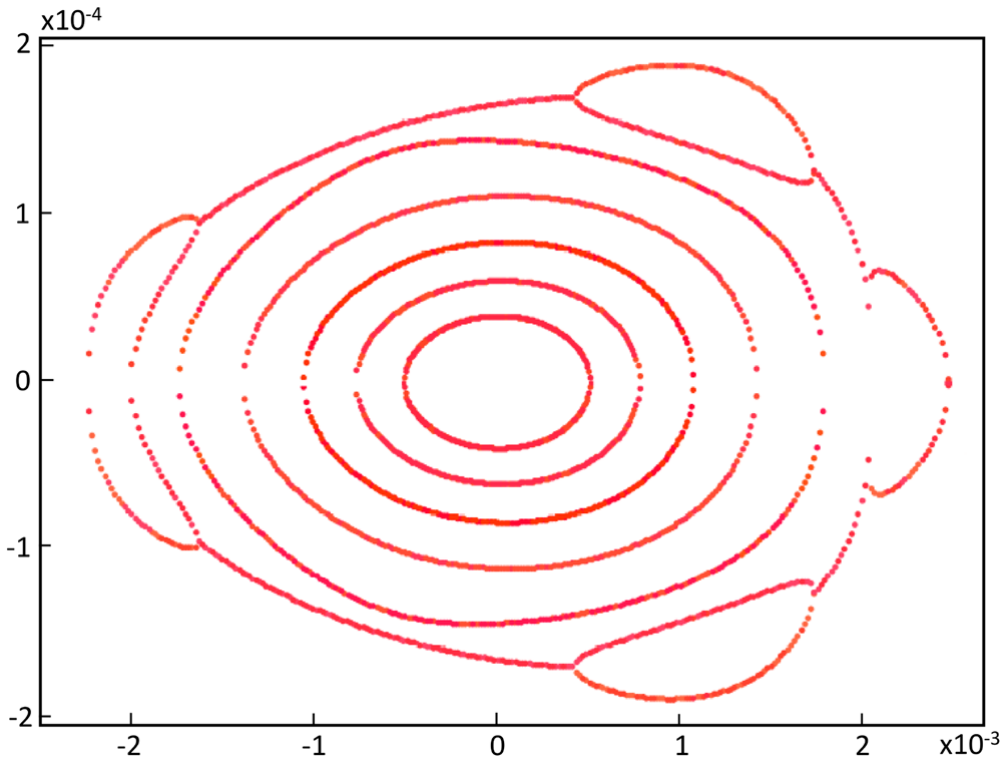
Horizontal phase space $(x,p_x)$ for the initial set of sextupoles $\{S_0\}$ given by Eq. (25). The used amplitudes $x_0$ are 0.5, 0.75, 1.0, 1.25, 1.45, 1.53 mm. Only representative resonances and internal tori are depicted. Units in the plot are m-rad.

Figure 3 represents the horizontal phase corresponding to the initial set $\{S_0\}$, showing the resonances arising at an oscillation amplitude of $x_0=1.53$ mm.

The location of these sextupoles in the unit cell of the synchrotron toy model considered here can be seen in Fig. 2, in green.

The initial $\{S_0\}$ free values can in general be selected as a set of small, arbitrary sextupole intensities, and the $x_0$ initially must be small enough to contain a stable zone. By increasing $x_0$, the optimization yields new sets of sextupoles with larger stable zones.

### Second stage: sextupoles optimization

**Figure 4:**
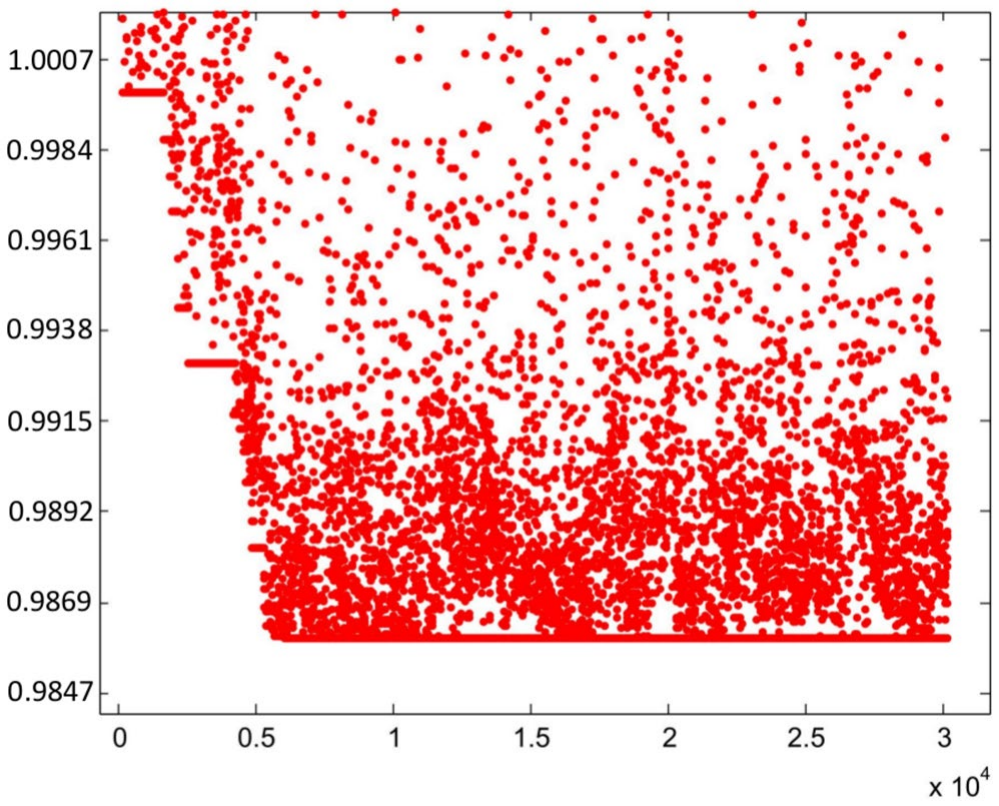
This figure shows the behavior of the objective function $f_{obj}$ defined in Eq. (21) as the number of iterations increases. The vertical axis represents the relative value of the objective function with respect to its initial value $f^{initial}_{obj}$, and the horizontal axis represents the cumulative CPU time (in seconds) as iterations proceed.

The optimization process was started with a small $$x_0$$ since the non-linear effects produced by the sextupoles are small. Within the optimization process, as $$x_0$$ grows, the algorithm seeks the trajectories in phase space to be stable. In this stage, starting from the set of sextupoles $$\{S_0\}$$, the search for a set of sextupoles that increases the previous dynamic aperture begins; here the oscillation amplitude is taken to be $$x_0=7$$ mm. This is done using genetic algorithms^45^ trying to minimize the objective function $$f_{obj}$$. Other methods, such as simplex of the $$\hbox {MATLAB}^{\circledR}$$ function *fminsearch* and simulated annealing, could also be useful. This process is shown in Fig. 4, where the vertical axis is the relative value of $$f_{obj}$$ with respect to its initial value $$f^{initial}_{obj}$$. It is convenient to note that in the optimization process of the sextupoles, the interval of values of the objective function is (0.9859, 66.432). However, only the range of interest ($$f_{obj}/{f^{initial}_{obj}}) < 1$$,where the objective function decreases, is shown in Fig. 4. The apparently small improvement is due to the fact that the initial $$\{S_0\}$$ was a set of sextupoles that already gave a small value of the objective function. The horizontal axis is the cumulative CPU time (in sec) of the genetic algorithm on a Pentium7 PC. The number of populations involved in the calculation was 14, and the process converged in around 1000 generations (Fig. 4).

As has been studied in detail in reference^26^, the resonances that appear in Fig. 3 arise when two branches of real solutions, from the quasi-invariant (Eq. (9)) proposed for the nonlinear case, overlap becoming complex solutions. The non-overlapping regions form the islands of the resonances. When the optimization process starts, in search of new sextupoles that allow increasing the dynamic aperture, the mentioned branches of real solutions are affected due to the numerical change of $\{S\}$ values. Minimization of function $f_{obj}$ (Eq. (21)) requires that the inner branch of real solutions be close to the corresponding solution of the linear problem (ellipse). This behavior occurs when the dynamics of the nonlinear problem allows an increase of dynamic aperture; otherwise, the roots overlap, generating resonances that limit stability.

**Figure 5:**
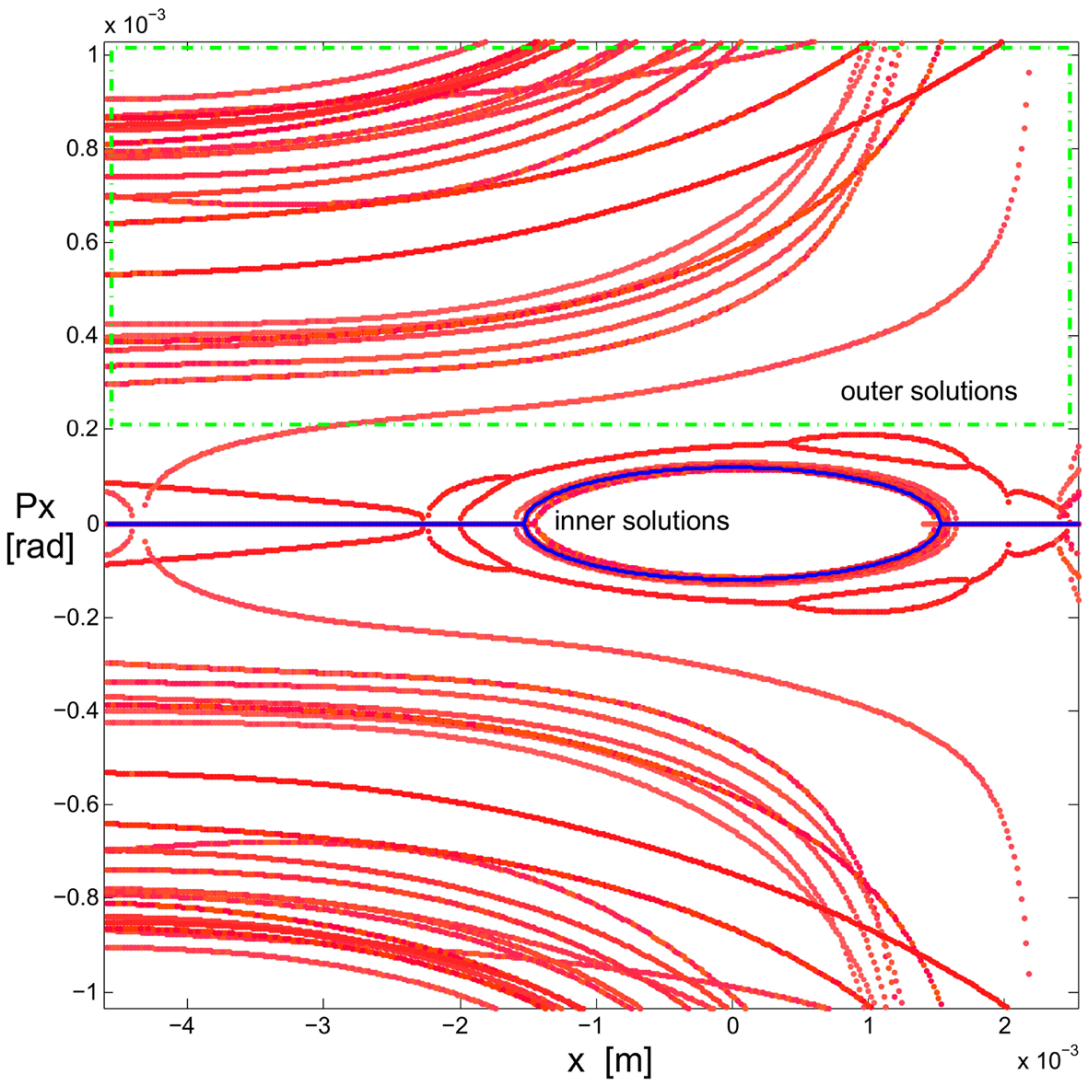
Phase space $(x,p_x)$ showing the evolution of the two real solutions (in red), under the optimization process of the objective function (when $\{S\}$ values change). The ellipse, corresponding to the Courant-Snyder invariant of the linear problem, is shown in blue for comparison; it is the optimization method goal. The horizontal blue lines for $p_x$ = 0 depict the real part of $p_x$, while the imaginary part is not shown in the figure, i.e., $p_x$ is a purely imaginary number. The outer branches are located mainly in the green rectangle, and they correspond to several $\{S\}$ values for the single invariant used in the optimization process of the second stage (section III.E).

**Figure 6:**
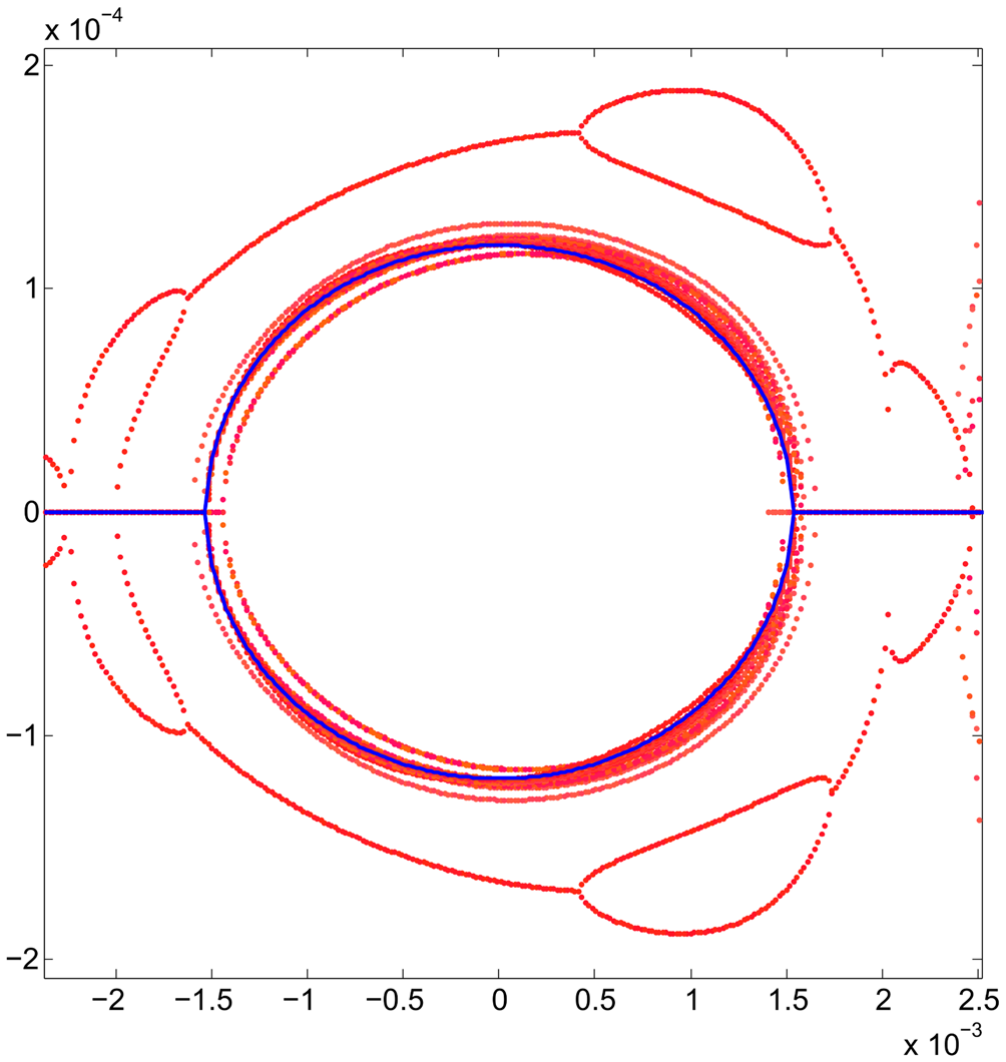
Phase space $(x,p_x)$ showing the original resonances and, in greater detail than in Fig. 5, the behavior of the inner real roots (in red) trying to fit the ellipse (blue) of linear dynamics, under the optimization process of the objective function. Units in the plot are m-rad.

Figure 5 shows the behavior of these two branches of real solutions (in red), as the values of *S* change. The external branch, contained mainly in the green rectangle, has a more erratic behavior than the internal branch and, in general, the external branch separates from the internal one under the optimization process. It is expected that the farther away a polynomial solution is from the origin of the phase space, $p_x$ will have a higher degree of uncertainty. Particle tracking simulation will show the agreement when comparing the results obtained with the proposed method with simulation, even for large oscillation amplitudes, as shown later. The linear-problem ellipse is used as a goal of the optimization method. It can be seen that the inner branch of real solutions tends to approach the blue curve that represents the trajectory corresponding to the Courant-Snyder invariant for the linear problem^27^. The latter can be appreciated in greater detail in Fig. 6 where a band of real solutions in red can be seen next to the blue ellipse. At the end of the optimization process, the real nonlinear solution, which is closest to the linear solution, is the one with a minimum value of $f_{obj}$. This will become clearer in the next stage of optimization.

**Figure 7:**
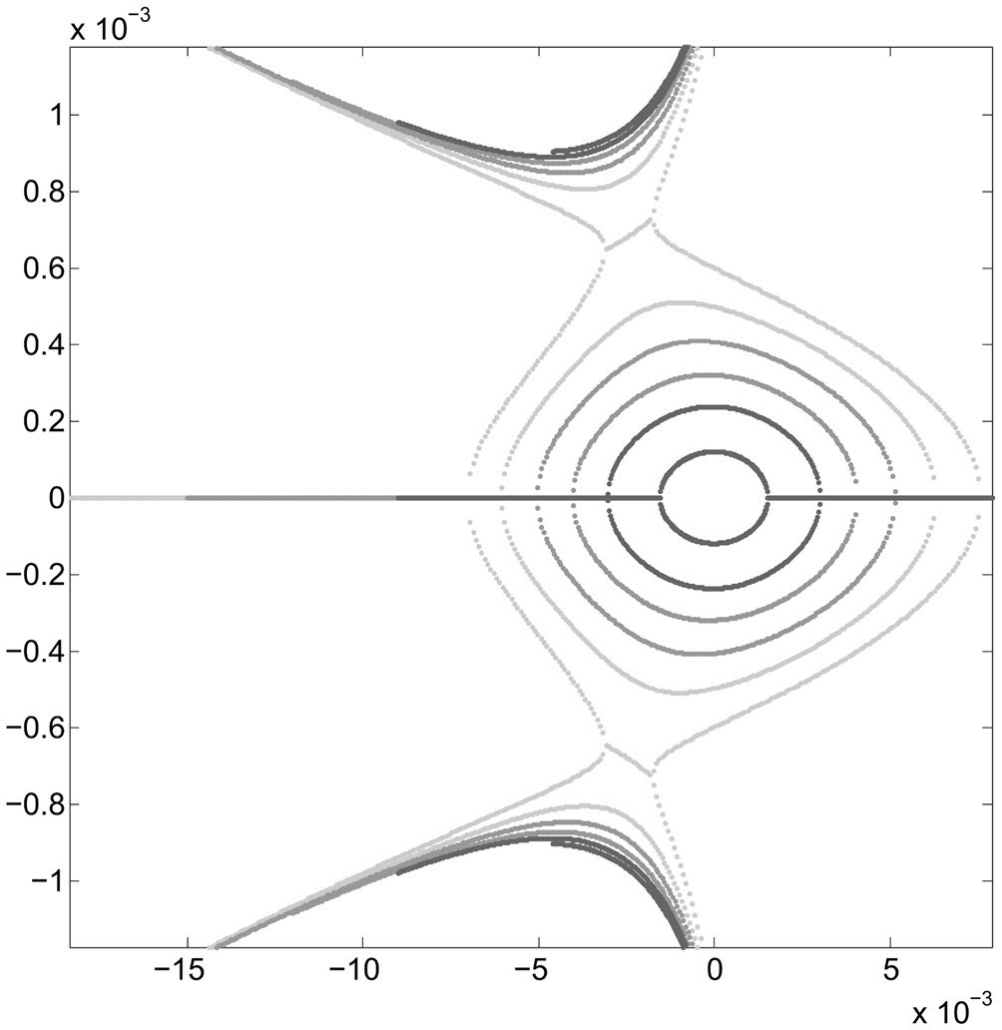
Phase space $(x,p_x)$ for various oscillation amplitudes $x_0=1.53, 3, 4, 5, 6, 7$ mm. With the new sextupoles $\{S_1\}$, obtained by optimizing to $x_0=7$ mm, the dynamic aperture is explored and it is observed that it increases to $\sim 6-7$ mm, before tori start to break. For each value of the quasi-invariant, the inner branch and its corresponding outer branch are plotted in the same grayscale color. Units in the plot are m-rad.

The above procedure allows to find the value of new sextupoles $\{S_1\}$ that minimize $f_{obj}$ as shown in Fig. 4. The new $\{S\}$ values are:

26 $$\begin{aligned} \{S_1\}=&\{1.4659108229654287, -1.6645577302767018,\nonumber \\ {}&-8.9509019610107163, -15.441140170269934,\nonumber \\ {}&17.416398341152696, 6.4256740018934524,\nonumber \\ {}&10.142133777479255, -22.142621628627953,\nonumber \\ {}&15.173009378441337\}, \end{aligned}$$

where the order of $\{S_1\}$ values is the same as that used in Eq. (24). As before, for each free parameter choice $\{S_1\}_{free}$, two chromatic sextupoles $\{S_1\}_{chrom}$ that allow keeping the chromaticities close to zero are determined. The following section addresses the implications of a new set of sextupoles $\{S_1\}$ in the nonlinear dynamics described in phase space ($x,p_x$). When this set of sextupoles is used, the phase space of Fig. 7 shows that the region of stability grows from $\sim 2$ mm to $\sim 6-7$ mm, since low order resonances onset have been inhibited in that region.

### Third stage: Further optimization of sextupoles

Now, we are exploring the possibility of continuing to grow the stability zone with a new optimization of sextupoles. This stage is carried out at a higher oscillation amplitude, $$x_0=10$$ mm, taking $$\{S_1\}$$ as the initial set of sextupoles for this stage of the optimization process, under the same guidelines as the previous optimization at $$ x_0=7$$ mm.

**Figure 8:**
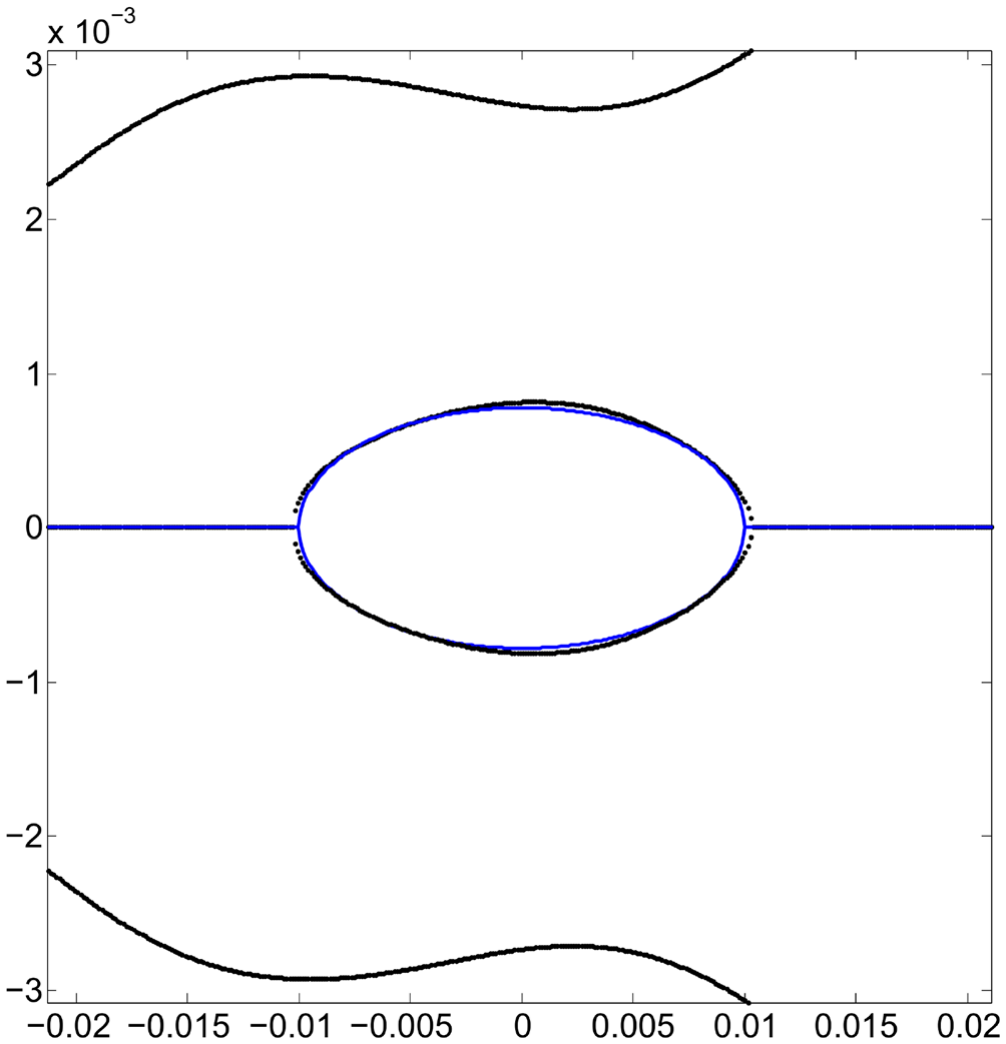
Structure of the phase space $(x,p_x)$ at the end of the third optimization process stage of the sextupoles set. The black curves are obtained as the branches of real roots solutions that give a minimum value of $f_{obj}$. Starting from the sextupoles $\{S_1\}$, that had optimized the objective function $f_{obj}$ with amplitude $x_0=7$ mm, a new set of sextupoles $\{S_{2}\}$ is found when $x_0=10$ mm is used. With the new sextupoles $\{S_{2}\}$ (Eq. (27)), no low order resonances are present since the real solution branches have been separated by the optimization process when requested that the inner branches are close to the Courant-Snyder ellipse, in blue. The dynamic aperture is now beyond 10 mm. Units in the plot are m-rad.

**Figure 9:**
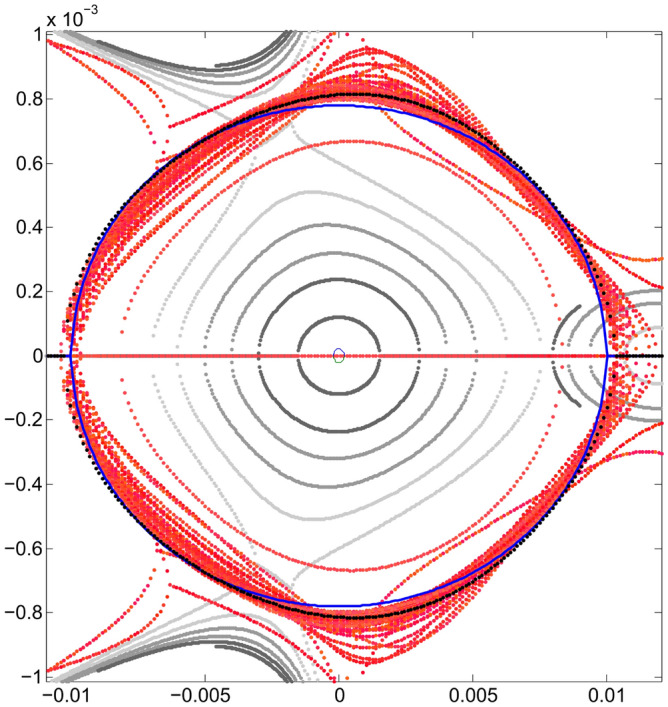
Enlargement of the phase space $(x,p_x)$ of Fig. 8 that depicts the set of inner branches (red) of the nonlinear problem in greater detail. These are intermediate solutions in the optimization process, from which the sextupoles, that minimize the objective function $f_{obj}$, arise. With the new sextupoles $\{S_{2}\}$, obtained by optimizing at 10 mm, we see that the estimated dynamic aperture (black) has grown beyond 10 mm. The black solution is the best approximation to the ellipse (blue) that represents the linear solution, according to the Courant-Snyder invariant. The gray tone curves show the structure of the underlying phase space (Fig. 7) before doing the new optimization using $x_0=10$ mm.

The inner and outer branches of real solutions that give a smaller value of the objective function $f_{obj}$ are shown in black in Fig. 8. They represent the final result of the optimization for the invariant corresponding to $x_0=10$ mm. Two of the branches of real solutions (inner branches) are close to the Courant-Snyder invariant represented by the blue ellipse. The other two branches (outer branches) are now far from the inner ones, meaning that, the possibility to generate low order resonances, by overlapping, has been diminished by the optimization process, increasing in this way the stability zone due to a better selection of sextupoles set. We see that the internal black branches resemble an ellipse, as requested in Eq. (21) of $f_{obj}$. The detail of this behavior is clearer in Fig. 9, where a bundle of intermediate solutions of the nonlinear problem in the optimization process is shown in red. The black solution is the best approximation to the linear solution in blue. The set $\{S_2\}$ of sextupoles obtained at the end of this stage is

27 $$\begin{aligned} \{S_2\}=&\{-12.652556508323647, -14.064768921050906,\nonumber \\ {}&-9.3690097475880325, -13.208398288099152,\nonumber \\ {}&13.876702657250986, 9.9207821018681628,\nonumber \\ {}&9.0521585477227173, -23.680418521419298,\nonumber \\ {}&12.458479828590827\}. \end{aligned}$$

Again, for comparison, the gray curves shown in Fig. 9 are the ones shown in Fig. 7 for the optimization $\{S_1\}$.

**Figure 10:**
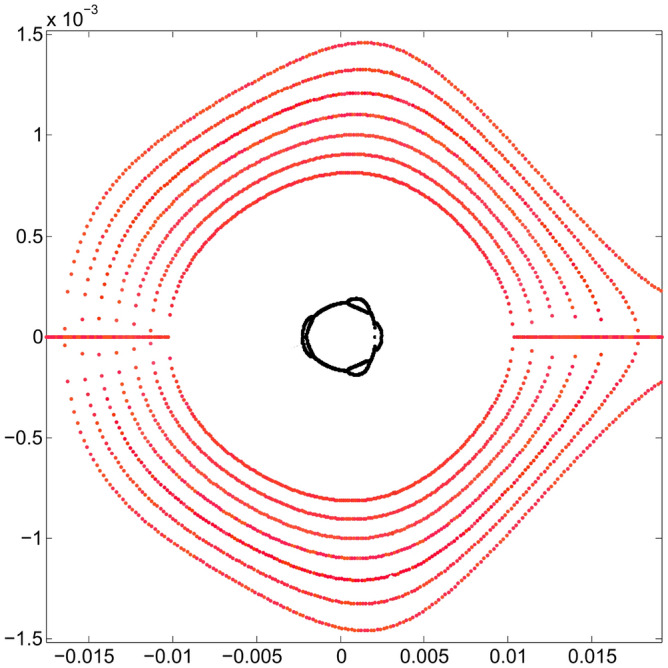
Structure of the phase space $(x,p_x)$ showing the dynamic aperture estimated by the quasi-invariant method, after optimization at $x_0=10$ mm, with the set of sextupoles $\{S_{2}\}$. The quasi-invariant method predicts stability for amplitudes of the order of 15 mm. For comparison purposes, the resonance structure (Fig. 3) provided by the initial set $\{S_0\}$ of sextupoles is included in black.

The next point of interest to investigate is the increase in dynamic aperture achieved with the $\{S_2\}$ optimization. For this, different values of oscillation amplitude $x_0$ are used that produce the trajectories shown in Fig. 10. We notice that closed trajectories start to break up at around 17 mm, although results coming from the quasi-invariant have been found to overestimate the dynamic aperture compared to that obtained by tracking simulation^26^.

### Comparison of dynamic apertures from quasi-invariant techniques with those from particle tracking simulations

**Figure 11:**
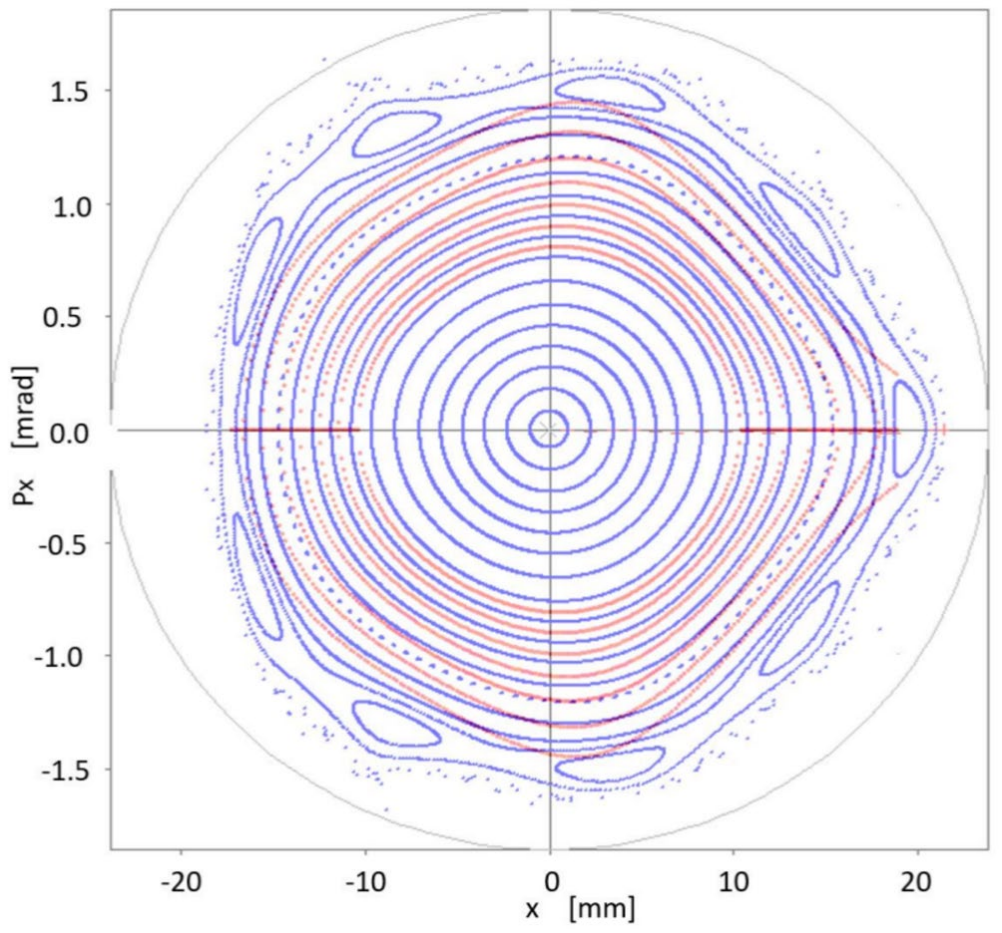
Structure of the phase space $(x,p_x)$ according to OPA particle tracking simulation (blue), showing good agreement with the dynamic aperture obtained with the quasi-invariant method (faint red superimposed, Fig. 10). Both calculations have been performed using the set of sextupoles $\{S_2\}$ for on-momentum particles ($\delta =0$).

How the theoretical results obtained with quasi-invariants compare with those of particle tracking simulation given by OPA? Using the set of sextupoles $\{S_2\}$ in OPA, the good agreement between both schemes is noteworthy. This is shown in Fig. 11, which represents the phase space ($x,p_x$) for on-momentum particles ($\delta =0$). In blue, several trajectories calculated with OPA, using different amplitudes, are shown. No low-order resonances are observed, which are a major problem in synchrotron light sources. The dynamic aperture shown in Fig. 11, of the order of 15 mm, confirms that the method of quasi-invariants gives a good prediction of the dynamic aperture. To make this agreement noticeable, the curves in Fig. 10 are superimposed in faint red.

### Optimization over on-momentum particles permeates to off-momentum particles

**Figure 12:**
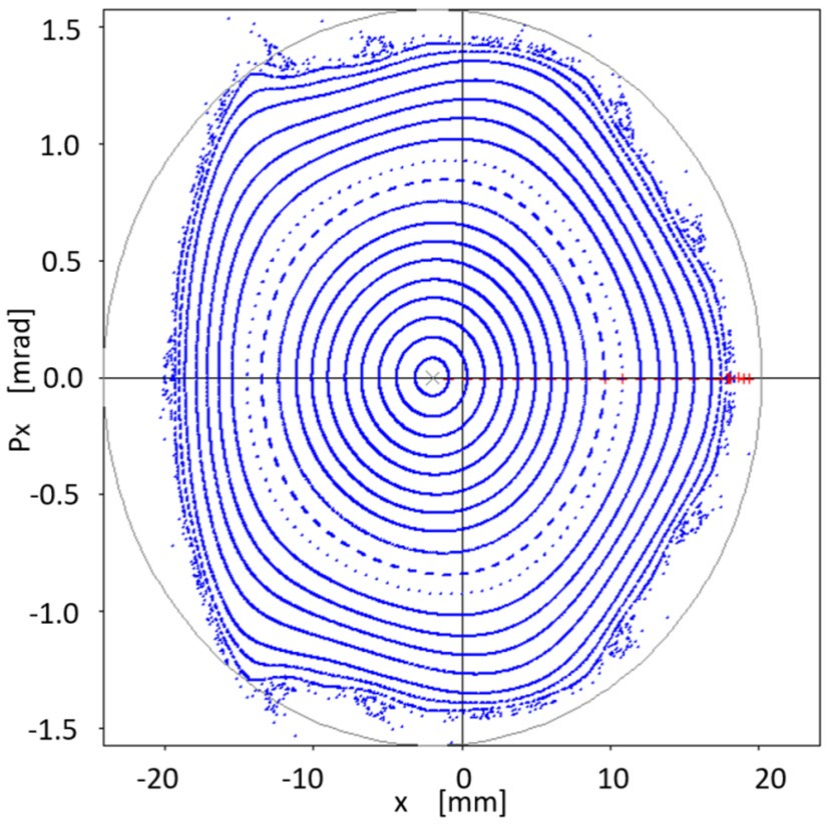
Structure of phase space $(x,p_x)$ for $\delta =-1\%$ when various initial conditions are used for particle tracking simulation in OPA, using the set of sextupoles $\{S_2\}$.

**Figure 13:**
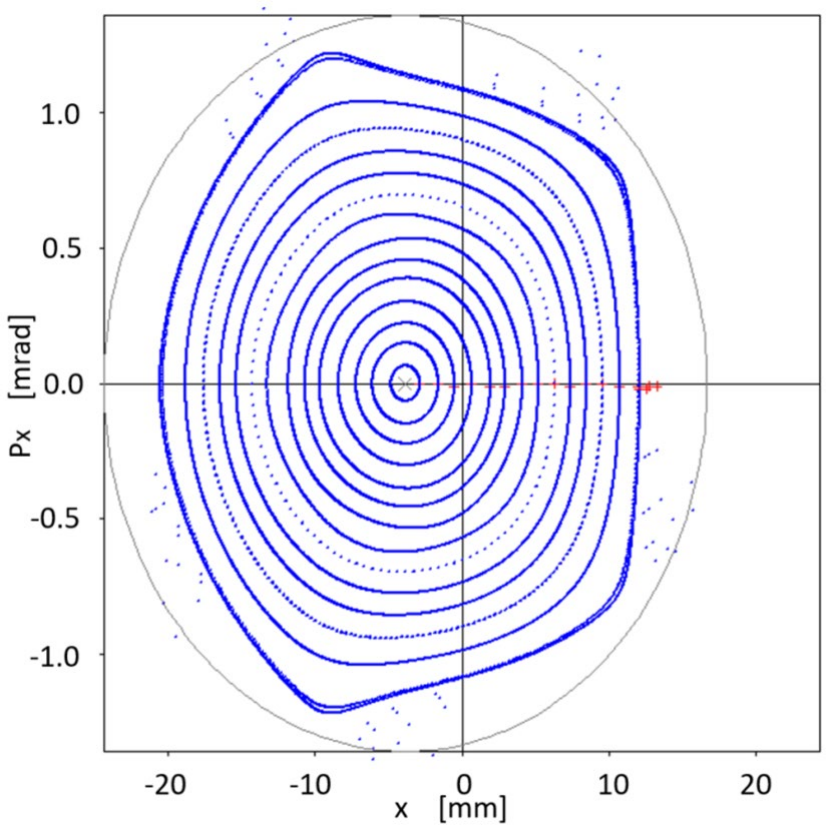
Structure of phase space $(x,p_x)$ for $\delta =-2\%$ when various initial conditions are used for particle tracking simulation in OPA, using the set of sextupoles $\{S_2\}$.

**Figure 14:**
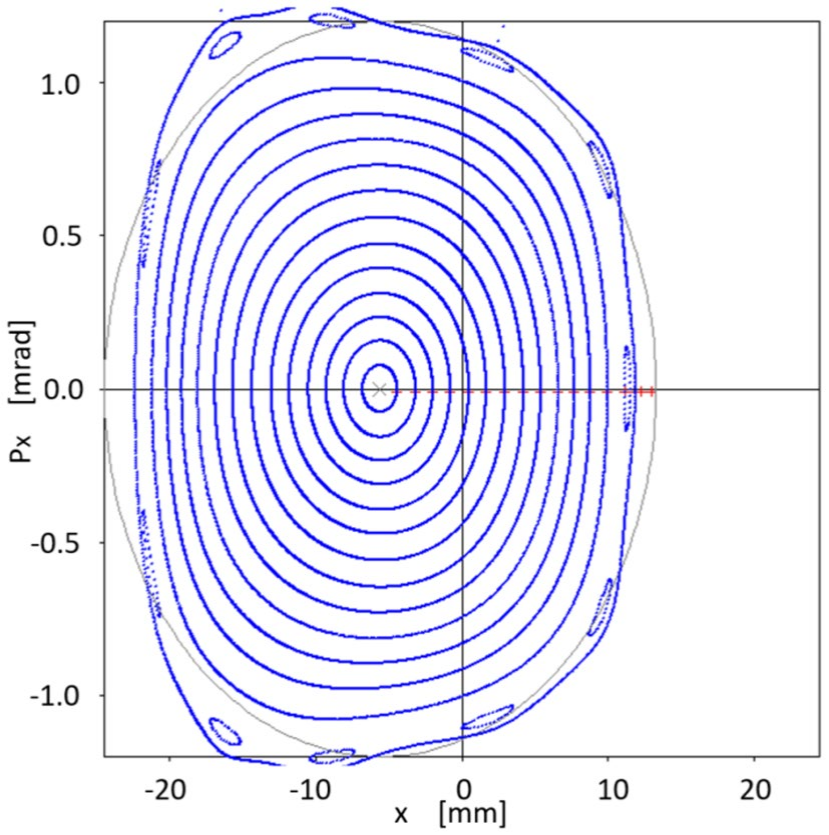
Structure of phase space $(x,p_x)$ for $\delta =-3\%$ when various initial conditions are used for particle tracking simulation in OPA, using the set of sextupoles $\{S_2\}$.

**Figure 15:**
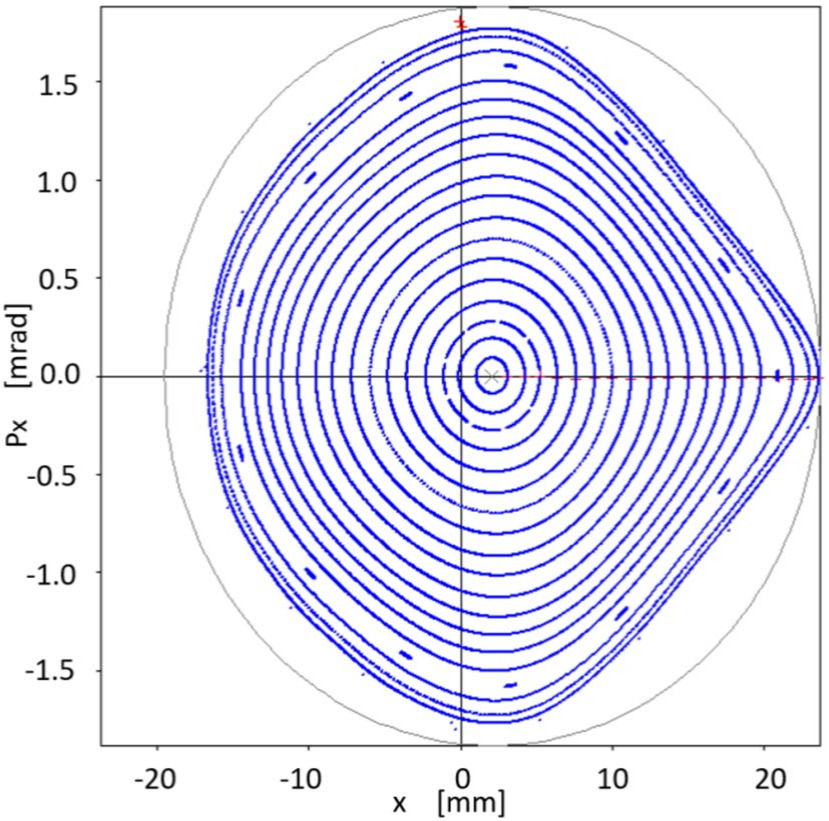
Structure of phase space $(x,p_x)$ for $\delta =1\%$ when various initial conditions are used for particle tracking simulation in OPA, using the set of sextupoles $\{S_2\}$.

**Figure 16:**
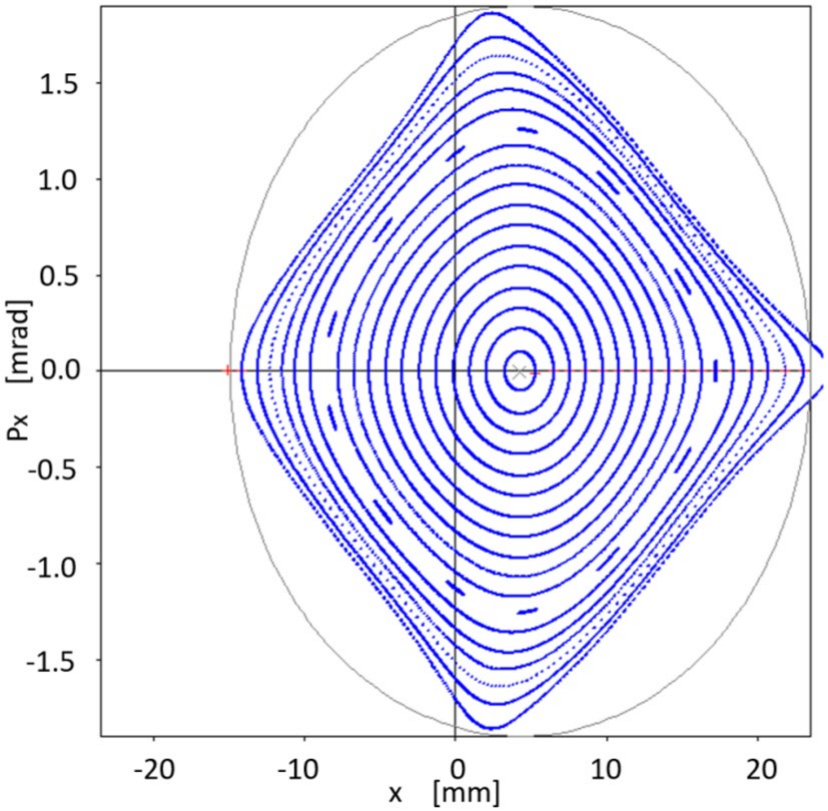
Structure of phase space $(x,p_x)$ for $\delta =2\%$ when various initial conditions are used for particle tracking simulation in OPA, using the set of sextupoles $\{S_2\}$.

**Figure 17:**
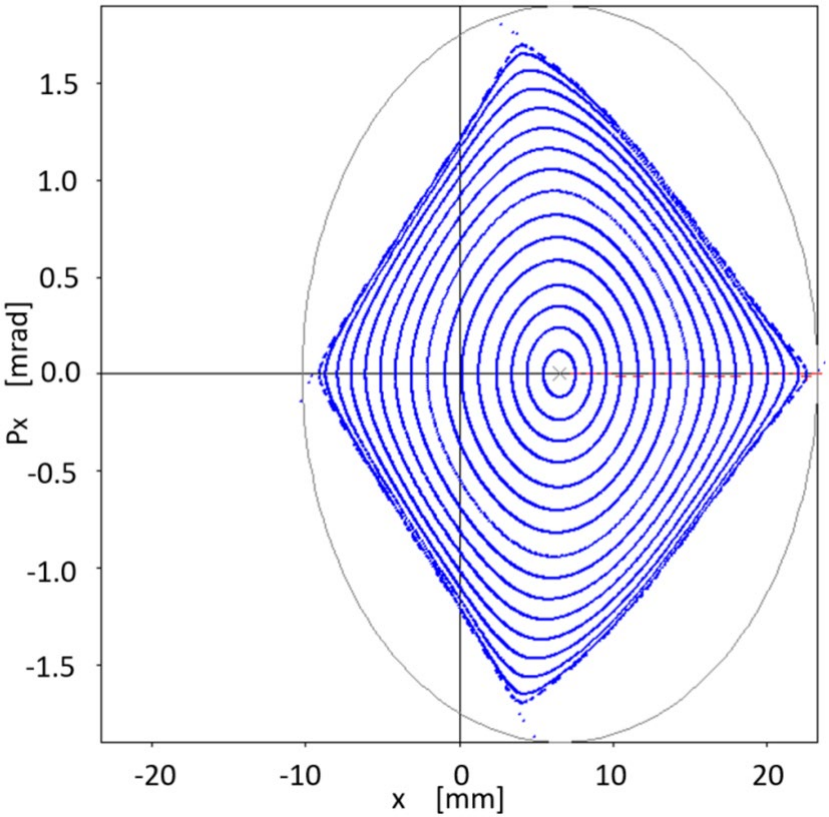
Structure of phase space $(x,p_x)$ for $\delta =3\%$ when various initial conditions are used for particle tracking simulation in OPA, using the set of sextupoles $\{S_2\}$.

Interestingly, although this work does not incorporate the formalism related to off-momentum particles $(\delta \ne 0)$, we see that the corresponding phase space, in this case provided by particle tracking simulation with OPA, also seems to follow the inertia of a linear dynamic, to which on-momentum particles have been forced with a small value of the objective function $f_{obj}$. That is, there is a certain degree of transmissibility of the stability of the considered phase space, when going from $\delta =0$ to $\delta \ne 0$. This process is observed in the following six figures (Figs. 12, 13, 14, 15, 16, 17), calculated with the set of sextupoles $\{S_2\}$, for a momentum deviation $\delta = -3,- 2,-1,1,2$ and $3 \%$. This is surprising because no optimization was done involving momentum deviation $\delta \ne 0$.

The origin shift in Figs. 12, 13, 14, 15, 16, 17 is consistent with the displacement of the off-momentum closed orbit given by $\eta \delta$ for a value of $\eta =0.2039$ m, which is the dispersion value in the straight sections for our toy model synchrotron.

### Limit on the oscillation amplitude for the optimization of the quasi-invariant

**Figure 18:**
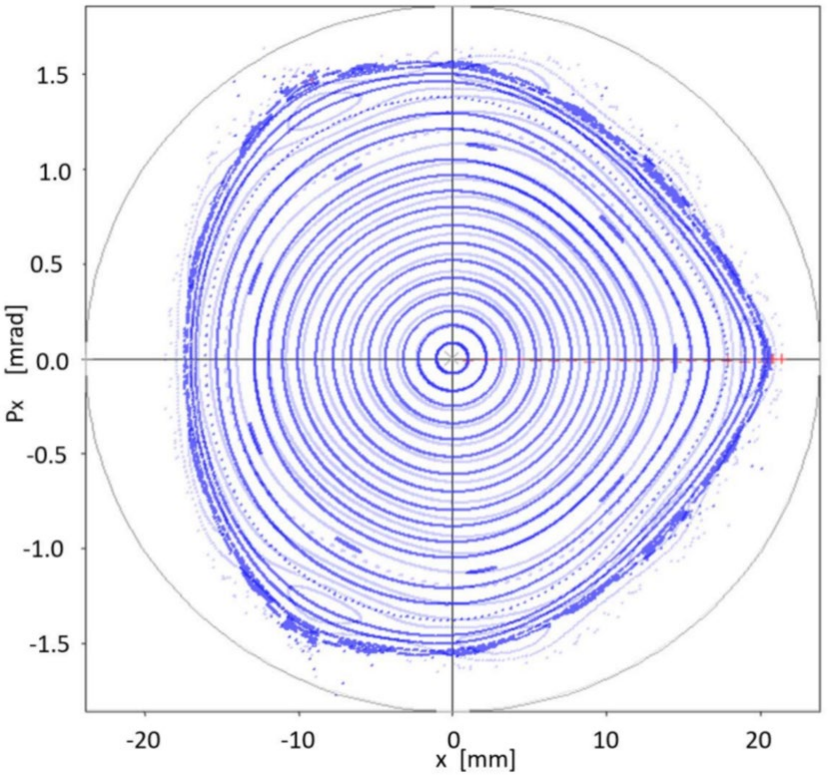
Structure of the phase space $(x,p_x)$ according to OPA’s particle tracking simulation (dark blue) done with the $\{S_3\}$ set of sextupoles obtained for a 12 mm amplitude optimization. In faint blue (background) the optimization at 10 mm of Fig. 11 is shown. In the new calculation (dark blue) there is a slight increase in the phase space area. Both structures (blue dark and faint) in phase space are for on-momentum particles ($\delta = 0$).

In previous sections phase space area results have been obtained by optimizations using the quasi-invariant defined in Eq. (9), carried out at amplitude values $x_0=7$ and 10 mm. What is being explored now is the possibility of further increasing the value of $x_0$. A new optimization is performed at $x_0=12$ mm and it is shown that the increase in dynamic aperture is insignificant, indicating that the non-linear dynamics of the considered synchrotron does not allow a greater increase in dynamic aperture, and/or the description of the quasi-invariant is less exact (the degree of the polynomial considered is not large enough) as the amplitudes $x_0$ increase. In Fig. 18 this process is shown. Optimization at 10 mm of Fig. 11 is shown in faint blue, while a new calculation (dark blue, superimposed) is obtained by the optimization process using an amplitude of 12 mm. Figure 11 was obtained with the set of sextupoles $\{S_2\}$, while this dark blue figure is made with the new set of sextupoles $\{S_3\}$, shown in Eq. (28), both, for on-momentum particles ($\delta =0$). Comparing both results, it is observed that the outer high order islands chain (faint blue) is inhibited. New KAM tori appear, marginally expanding the stable area of the phase space. It is interesting to note that the new selection $\{S_3\}$ differs less than $<2\%$ from $\{S_2\}$ which represents a fine adjustment of sextupole intensities. The effect is significantly smaller than that obtained going from 7 to 10 mm. This suggests that the optimization limit is being reached.

28 $$\begin{aligned} \{S_3\}=&\{-12.864277322994971, -14.050032324271484,\nonumber \\ {}&-9.3226604284599492, -13.099983409997680,\nonumber \\ {}&14.022145293554772, 10.011461482140758,\nonumber \\ {}&9.2036108097558724, -24.02863787948452,\nonumber \\ {}&12.21599140620257\}. \end{aligned}$$

For a synchrotron represented in one dimension, the results obtained are a clear indication of the robustness of the quasi-invariant concept and its use in increasing synchrotron dynamic aperture, requesting the topologies of the phase space of a nonlinear and a linear system to be similar in a bounded area of small oscillation amplitudes. The mathematical formalism of this idea can be extended to higher dimensions to achieve low emittance designs.

Although not analyzed in this paper, it is also noteworthy that studies using quasi-invariants report a large amplitude-dependent tune spread^20^. Therefore, it is expected that with our technique, resonance lines, probably of higher orders, can be crossed without noticeable effects due to their narrow stop bandwidths.

## Concluding remarks

This work has been motivated by the increasing requirements in optimizing the magnetic lattices of synchrotron light sources to meet the growing needs of users of synchrotron radiation, such as increased brightness and coherence. Methods developed by other authors have been essential to deal with the nonlinear problem, some of them involve a large capacity of computational resources. In this work, it is shown that the scheme based on a polynomial quasi-invariant is a dynamic aperture optimization technique complementary to methods using resonant terms or many turns particle tracking simulation^9^. The precision achieved with the quasi-invariants is sufficient to accomplish a dynamic aperture compatible with numerical tracking results. With these ideas, we introduce and explore one more mechanism useful to find solutions that allows advancing in the understanding of dynamic processes in synchrotrons. The proposed algorithm requires the construction of a quasi-invariant of motion whose use is valid in a restricted phase space area including its origin, where the electron beam should be stable. A quasi-invariant mechanism, and an objective function that allows manipulating the onset of resonances and, thereby, increasing the dynamic aperture of a particular synchrotron design, were studied. In the stability region, the resemblance between a non-linear system phase space topology and the one of the linear system, seems to be a key to achieve good results in increasing the dynamic aperture. The results obtained were validated by comparison with particle tracking simulations, using available software in the field of accelerator physics. The numerical results indicate that the proposed method can be used as a suitable scheme to increase the dynamic aperture in the one-dimension studied model. The methodology can be easily extended to two dimensions by building a second quasi-invariant, allowing to increase the dynamic aperture of the two-dimensional nonlinear problem, a phenomenon that is quite restrictive in fourth generation light sources. Work is ongoing in this direction.

## Data Availability

The data generated or analyzed during the current study are available from the corresponding author on reasonable request.
